# Supplementary material for: The default network dominates neural responses to evolving movie stories
Source: Nat Commun. 2023 Jul 14;14:4197. doi: 10.1038/s41467-023-39862-y (PMC10349102; doi:10.1038/s41467-023-39862-y)
Supplement: Supplementary file 1 — Supplementary Information [file 41467_2023_39862_MOESM1_ESM.pdf]

Supplementary material

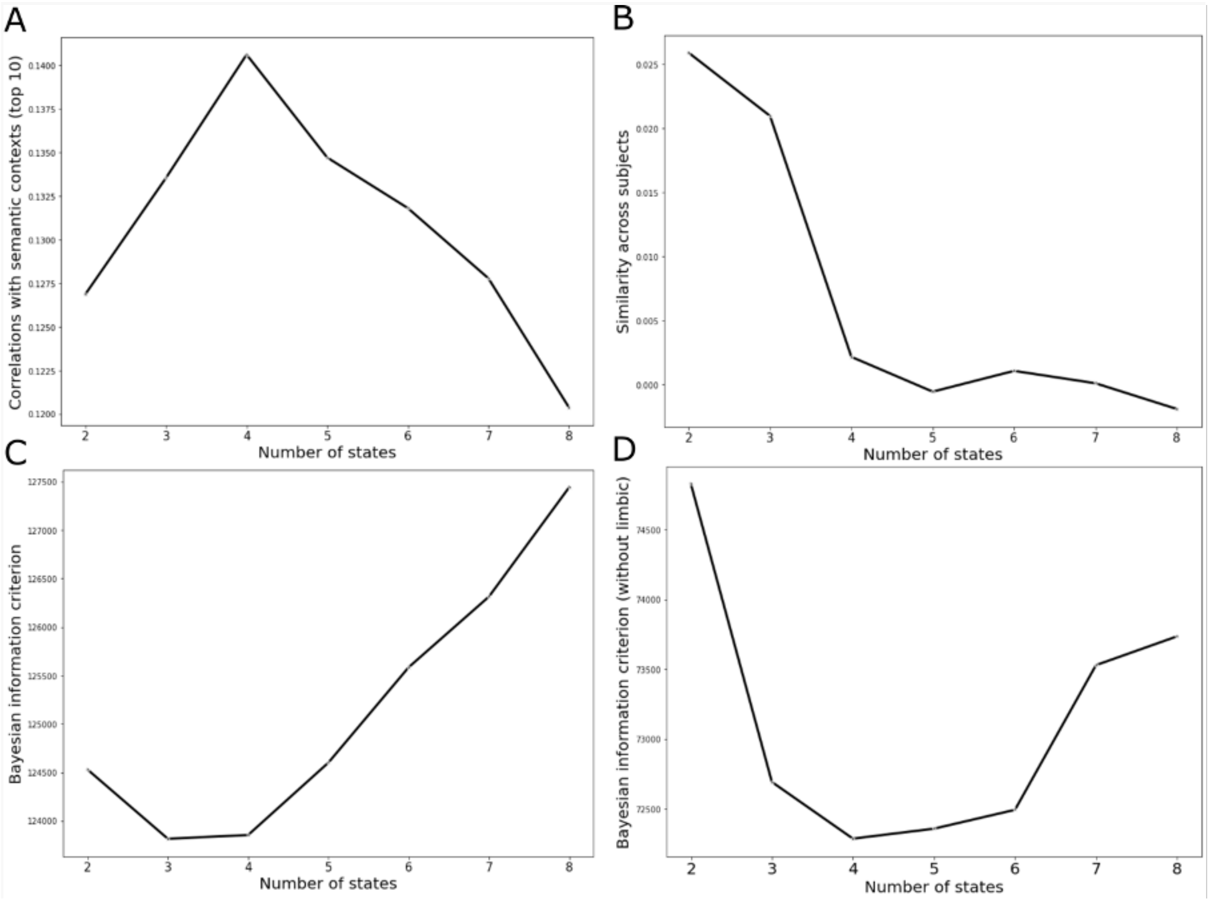

Supplementary Figure 1: Complementary metrics for HMM models converge to 4 state solutions. We justify the selection of 4 HMM states based on four complementary metrics. A) The average value of the top 10 Pearson correlation links between HMM models' state presence and extracted semantic contexts. The larger this value is, the closer the brain activity model aligned with movie narrative features. This value peaked for the choice of 4 states. B) The average Pearson correlations across 15 subjects' HMM state presence. The lower value represented more discovered dynamics. At the point of 4 states, this value decreased rapidly and fluctuated from 4 states to 8 states. C) Bayesian information criterion (BIC) measured the likelihood of the model while penalizing the model complexity. A lower value was preferred. This value reached its global minimum in the range of 3 to 4 states. D) We removed the effect of the limbic partners and evaluated BIC again. This value reached its lowest peak at the point of 4 states. Source data are provided as a Source Data file.

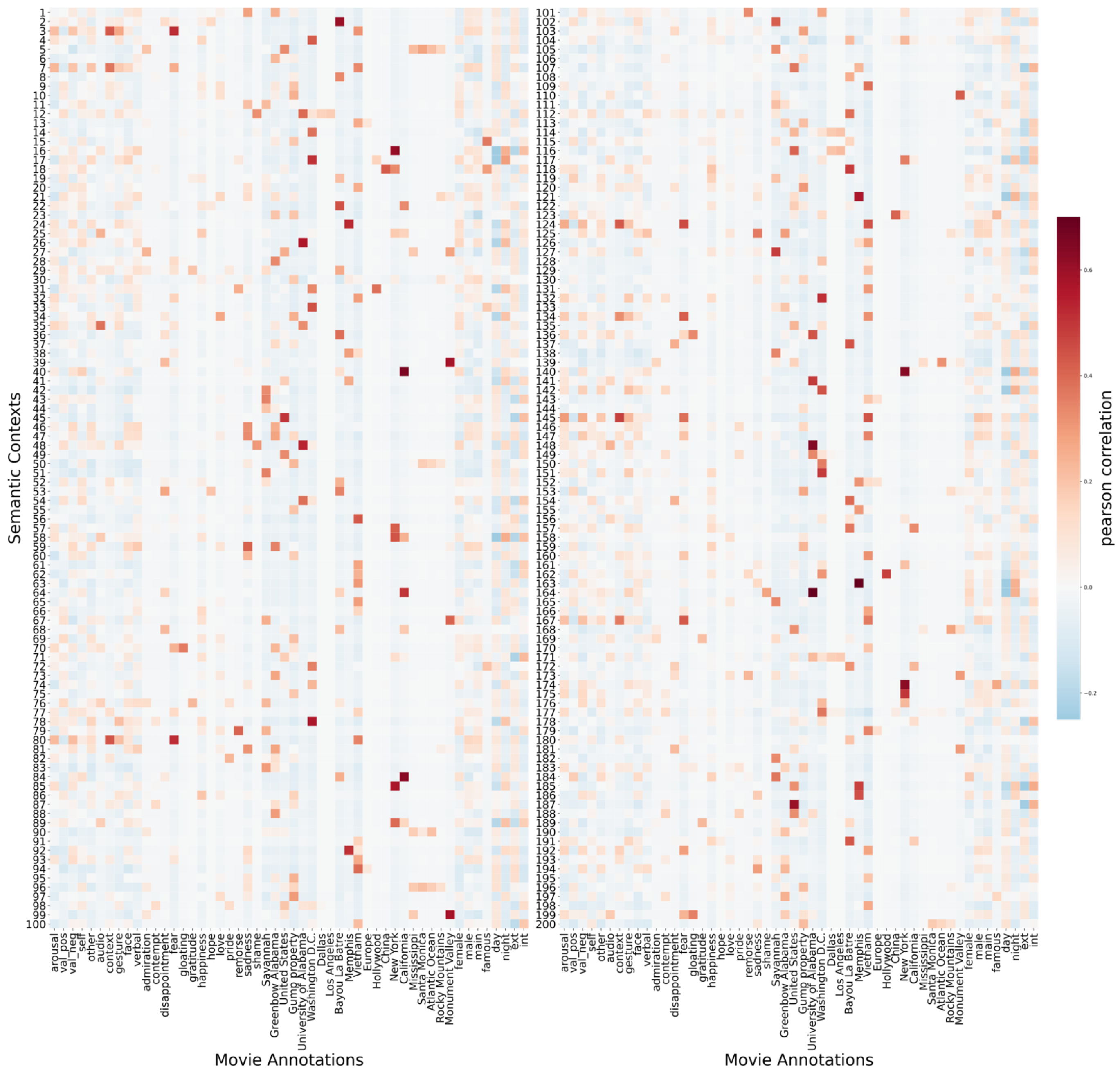

Supplementary Figure 2: The Pearson's correlations between 52 movie annotations and 200 semantic contexts. The left column showed the pairwise Pearson's correlation between 52 annotations and semantic contexts No. 1-No.100. The right column showed the correlations of the rest semantic contexts (No. 101 – No. 200). Several annotations (e.g., "fear") sparsely correlated with multiple semantic contexts, while many annotations generally had a low correlation value with most of the semantic contexts (e.g., "Europe"). Source data are provided as a Source Data file.

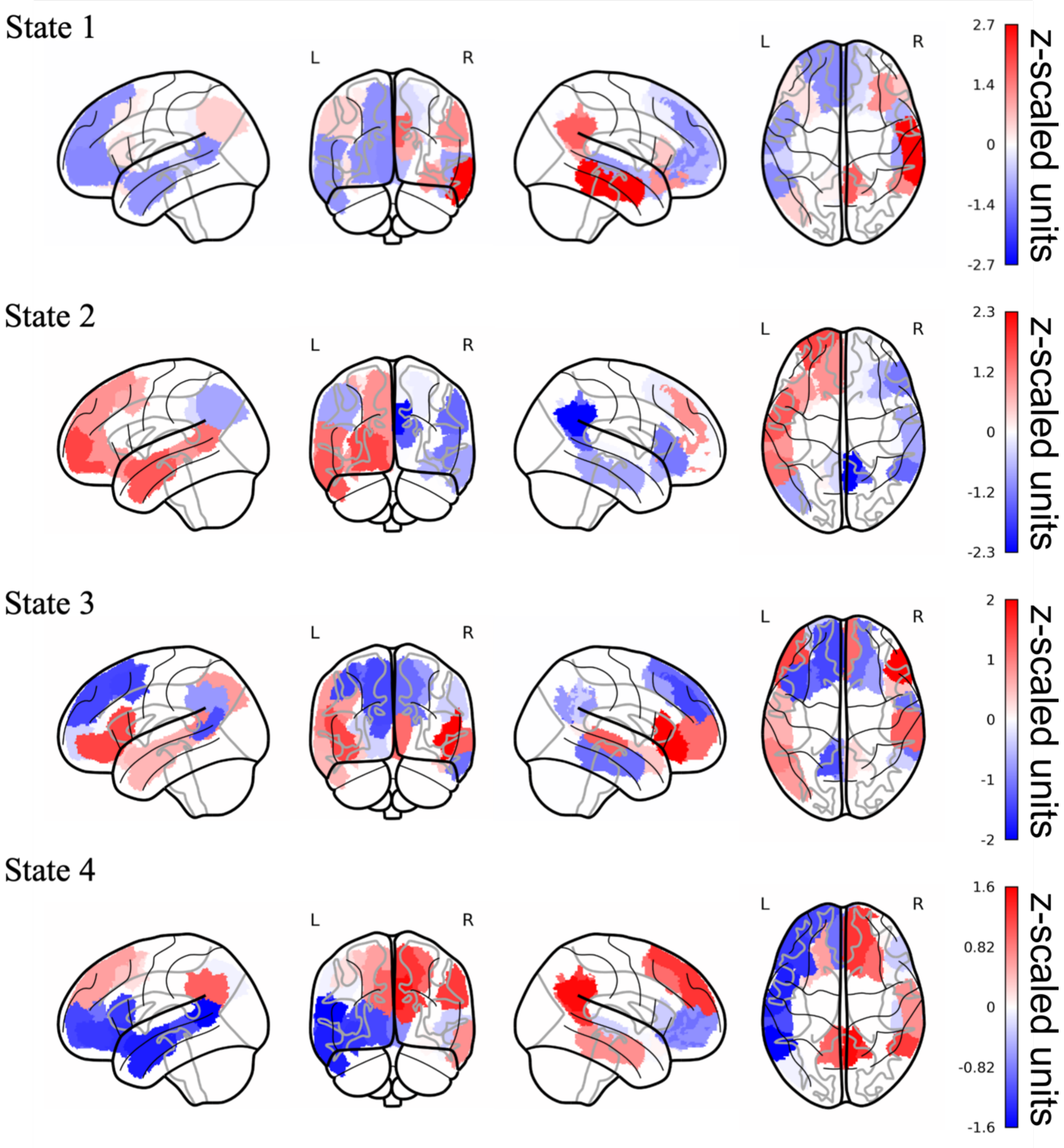

Supplementary Figure 3: Glass brain plots based on DN&AM region-network contributions for dynamic brain states 1-4. The strength of the color represented the mean contribution signatures of four states in subject1's DN&AM model. Source data are provided as a Source Data file.

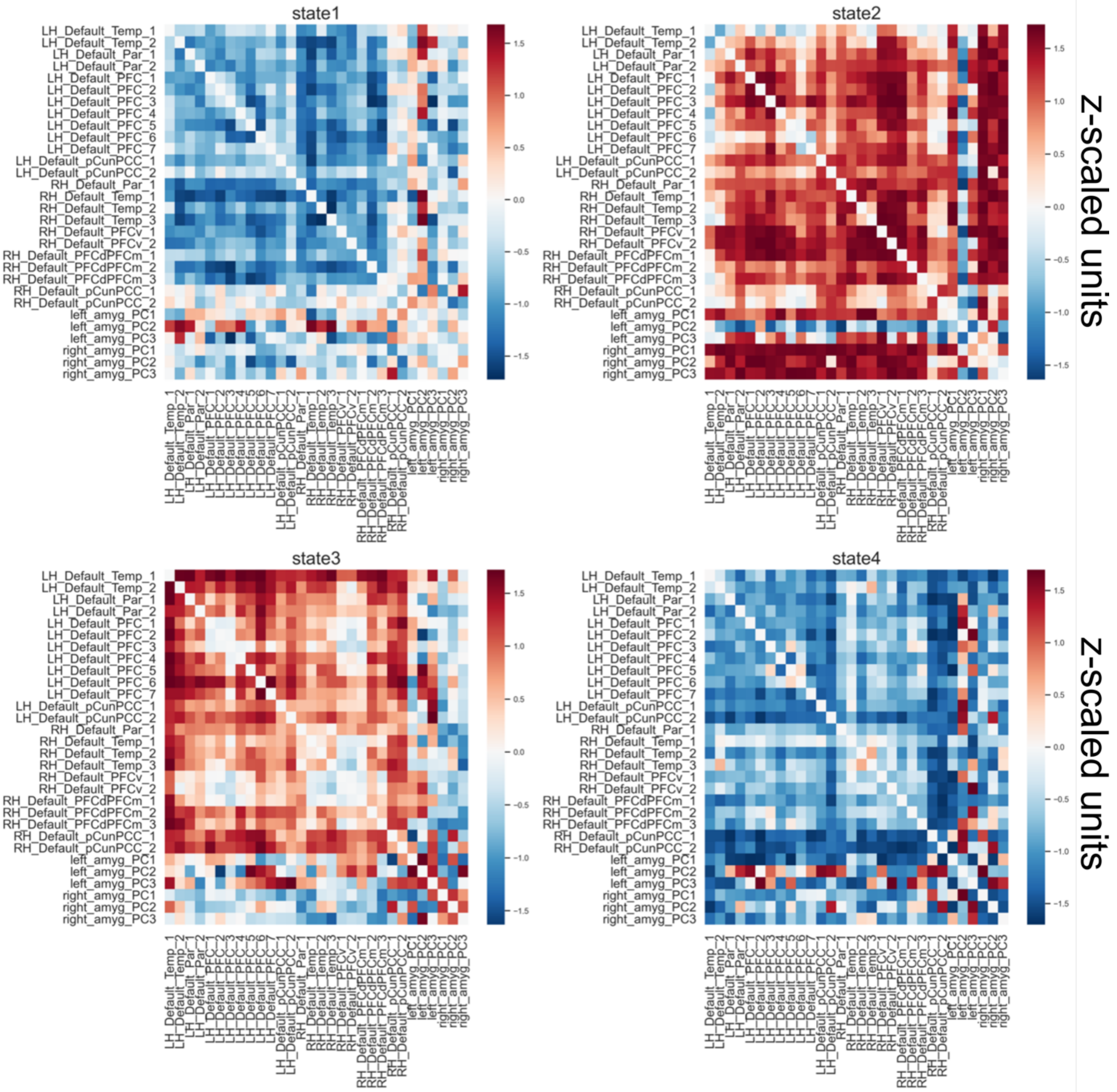

Supplementary Figure 4: The functional connectivity (FC) signatures of four states in subject1's DN&AM model. The four FC matrices were derived from the trained parameters of the multivariate Gaussian HMM model. The positive values (red) denoted that the two (sub)regions increase in the same direction, while the negative values (blue) indicated the two (sub)regions increase in the opposite direction. The DN's subregion names are from Schaefer Yeo 100 atlas (Temp: temporal; Par: parietal; PFC: prefrontal cortex; pCunPCC: precuneus posterior cingulate cortex; PFCv/d/m: ventral/dorsal/medial prefrontal cortex;). The bottom/rightmost 6 rows are principal components for the amygdala's left and right hemispheres. The left and right hemispheres are denoted as LH and RH. Source data are provided as a Source Data file.

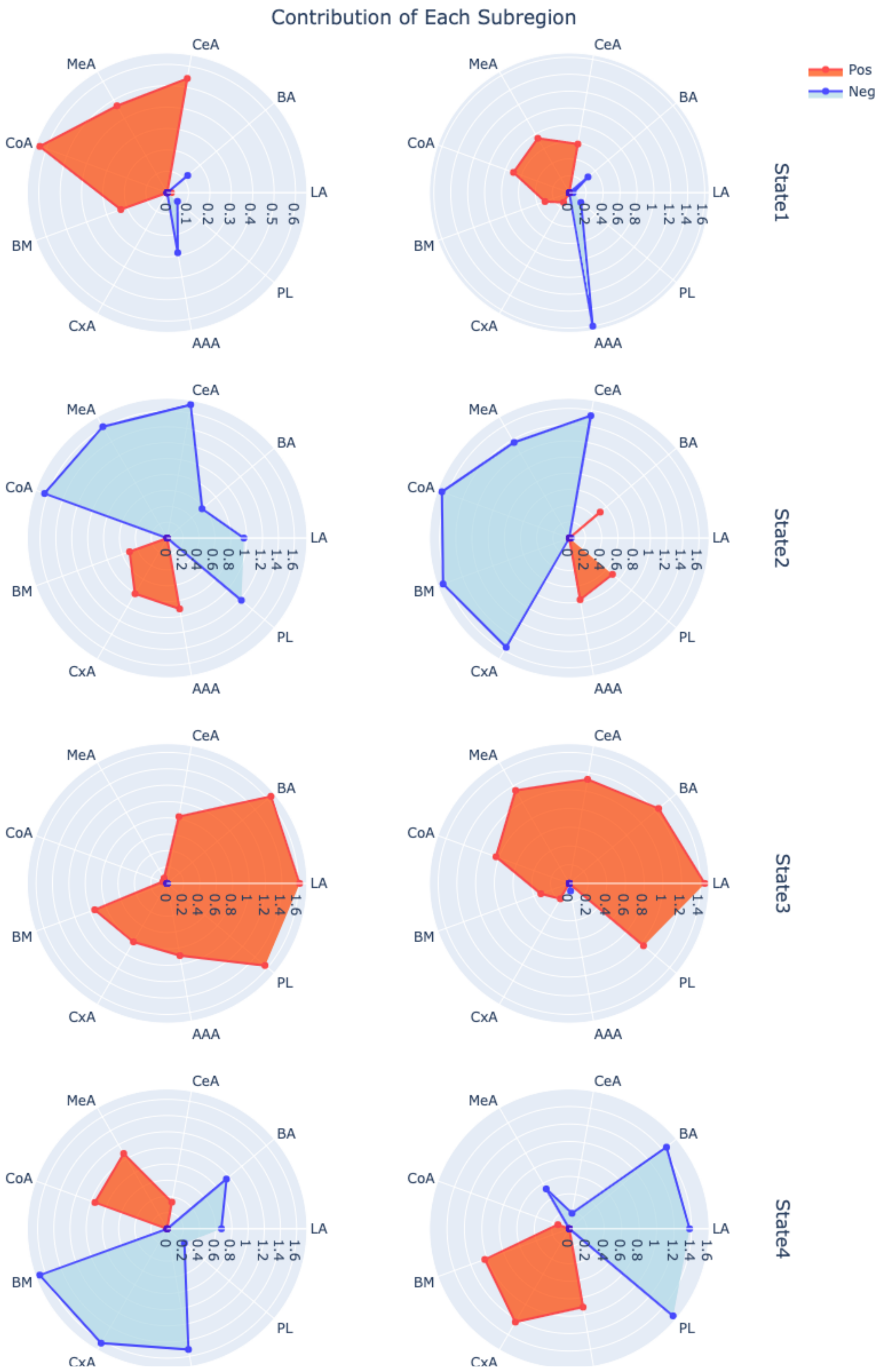

Supplementary Figure 5: The amygdala subregion signatures of four states in subject1's DN&AM model. Each row contained the the left and right hemisphere's signitures of one dynamic brain state. Great lateralization differences were observed in the amygdala subregions. LA: Lateral-nucleus; BA: Basal-nucleus; CeA: Central-nucleus; MeA: Medial-nucleus; CoA: Cortical-nucleus; BM: Accessory-Basal-nucleus; CxA: Corticoamygdaloid-transition; AAA: Anterior-amygdaloid-area; PL: Paralaminar-nucleus. Source data are provided as a Source Data file.

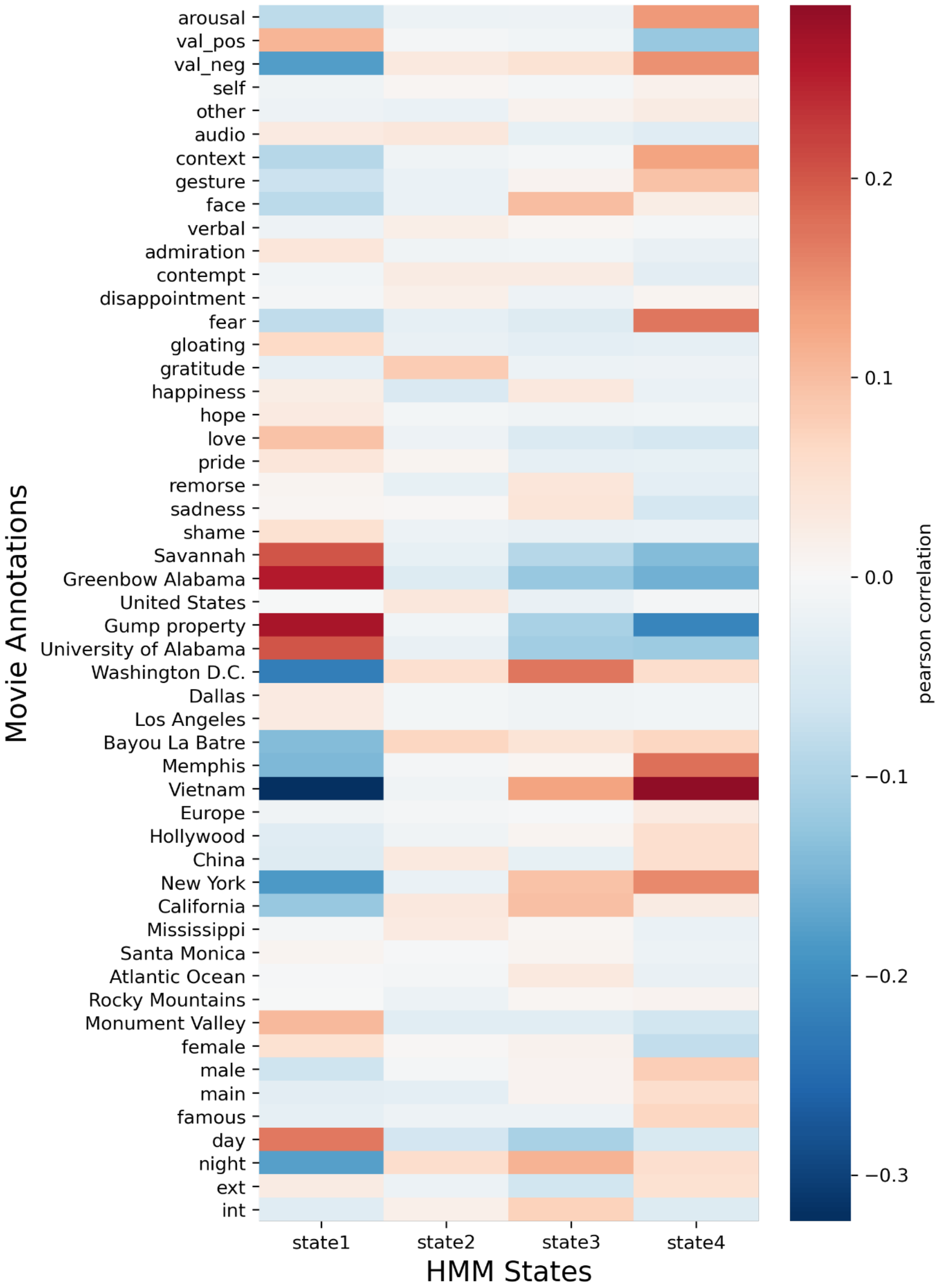

Supplementary Figure 6: Pearson's correlation between 52 human-curated annotations with four states of subject 1's DN&AM model. Pairwise pearson's correlation between 52 human-curated annotations and four dynamic brain states were shown in the heatmap. Each state's top correlated annotations had distinct profiles. Source data are provided as a Source Data file.

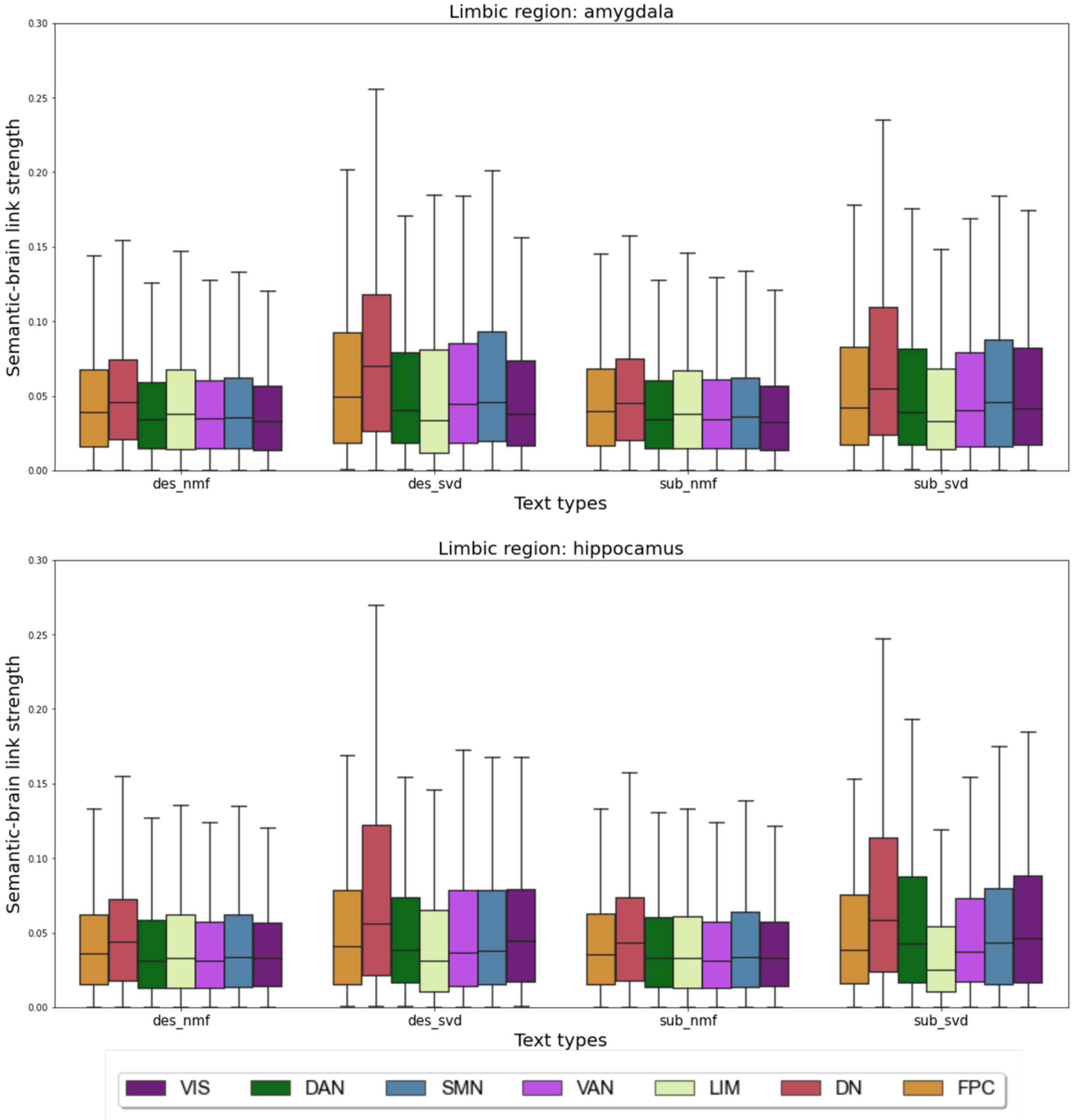

Supplementary Figure 7: Functional coupling dynamics across different text extraction methods and text sources. We performed several confirmatory analyses supplementing the main text's Fig. 4. These supplementary experiments aimed to analyze the robustness of the conclusion, that the DN tracks most of the semantic contexts. The top row showed the results paired with amygdala as the extra limbic region and the bottom one was with hippocampus. Inside the plot, each x tick denotes one unique sets of semantic contexts. The "des" represented that the text data is the human description of the movie instead of the subtitle (sub). The two different text extraction methods used for LSA (cf. methods) were non-negative matrix factorization (NMF) and singular value decomposition (SVD). We generated 200 semantic contexts via NMF-LSA and 5 semantic contexts via SVD-LSA. Thus, each box contains 12000 (200 semantic contexts x 15 subjects x 4 states) correlation values and 300 (5 sematnic contexts x 15 subjects x 4 states) for NMF-LSA and SVD-LSA respectively. Across different sets of semantic contexts, the median values of DN's semantic brain link strength were the largest. Boxplot: upper (lower) edge of the box is 25th (75th) percentile

(interquartile distance); the middle line is the median value; the green triangle shows the mean value; the whiskers summarize the extreme data points of the distribution of median semantics-brain associations. Short names for Schaefer-yeo networks: VIS: Visual network; SMN: somatomotor network; DAN: dorsal attention network; DN: default network; LIM: limbic network; VAN: salience and ventral attention network; FPC: Frontoparietal network. Source data are provided as a Source Data file.

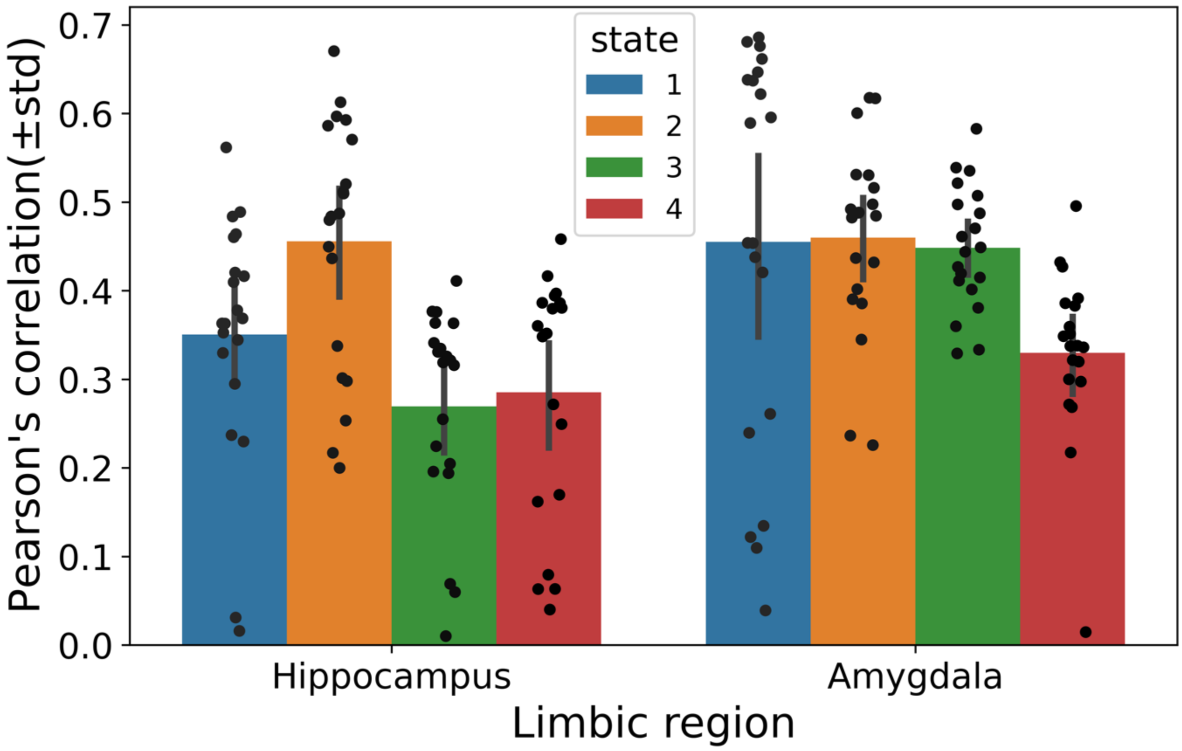

Supplementary Figure 8: 20-fold cross validation scores of the PLS-R projected embeddings. To test the generalization ability of current PLS-R analysis, we divided each subject's timeseries data of certain brain state into 20 different folds. Then, we trained the PLS-R model on the training set and test the Pearson's correlation of the projected BOLD embeddings and external label embeddings. The bar-plot showed the average correlation based on 20 different folds. State 2 of the DN&HC model had the largest r = 0.46, while state 3 of the DN&HC had the smallest r = 0.27. The error bar denotes 95% confidence interval. Source data are provided as a Source Data file.

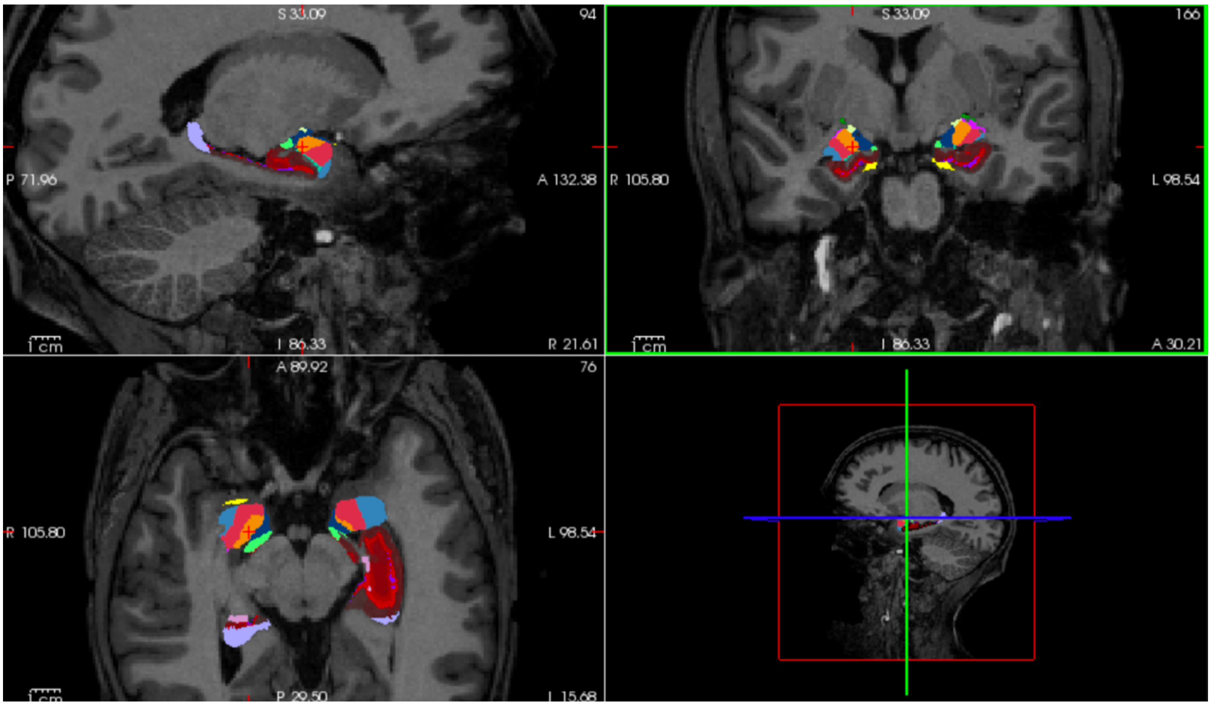

Supplementary Figure 9: The microanatomical segmentations generated from subject 1's structural MRI data via freesurfer. The shortcut from the freesurfer software delineated the hippocampus and amygdala segmentations.

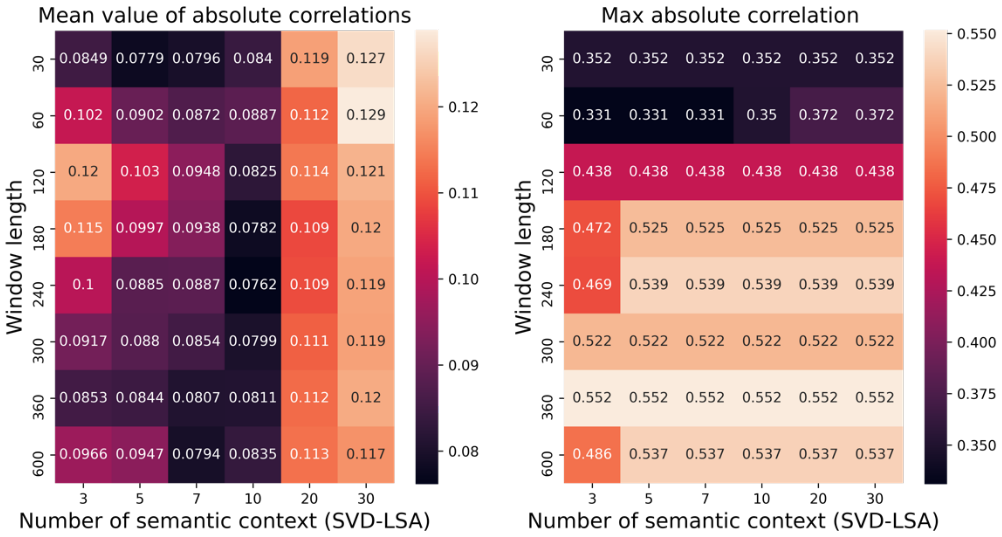

Supplementary Figure 10: The grid search of mean and max Pearson's correlation between semantic contexts and human-curated annotations. We performed a grid search to find the optimal window length to aggregate semantic information in the movie text data (cf. methods). Combining the performance of achieving both average and max correlations with annotations. The window length of 240s was the most optimal choice. Source data are provided as a Source Data file.

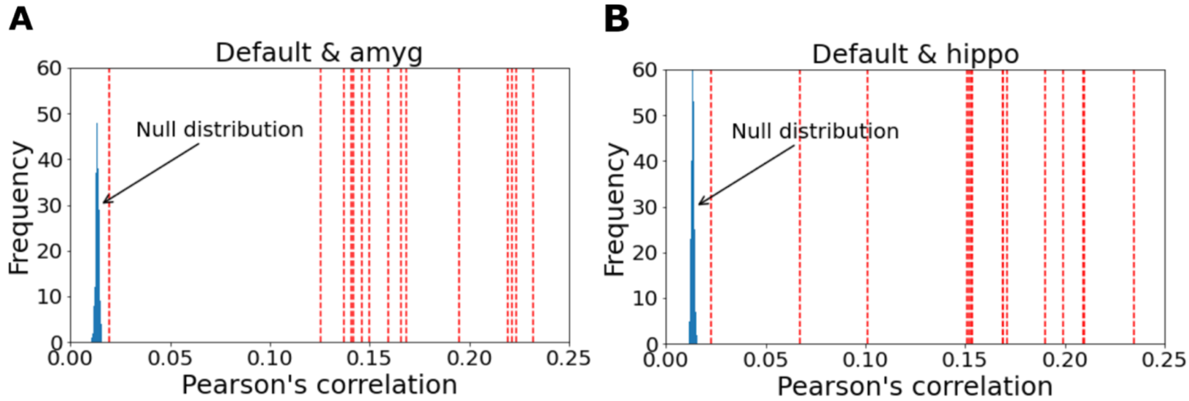

Supplementary Figure 11: Permutation test of the DN&AM and DN&HC semantic brain link strength. We used a permutation test to assess the significance of the correlation between the timeseries of BOLD signal of DN and our derived semantic labels. Specifically, we randomly shuffled the timeseries 1000 times and calculated the correlation coefficient for each shuffle. The results are summarized in the blue histogram. The red dashed line represents the correlation coefficient for the true data points of 15 subjects. A) Permutation test for DN&AM mode. B) Permutation test for DN&HC mode. Source data are provided as a Source Data file.

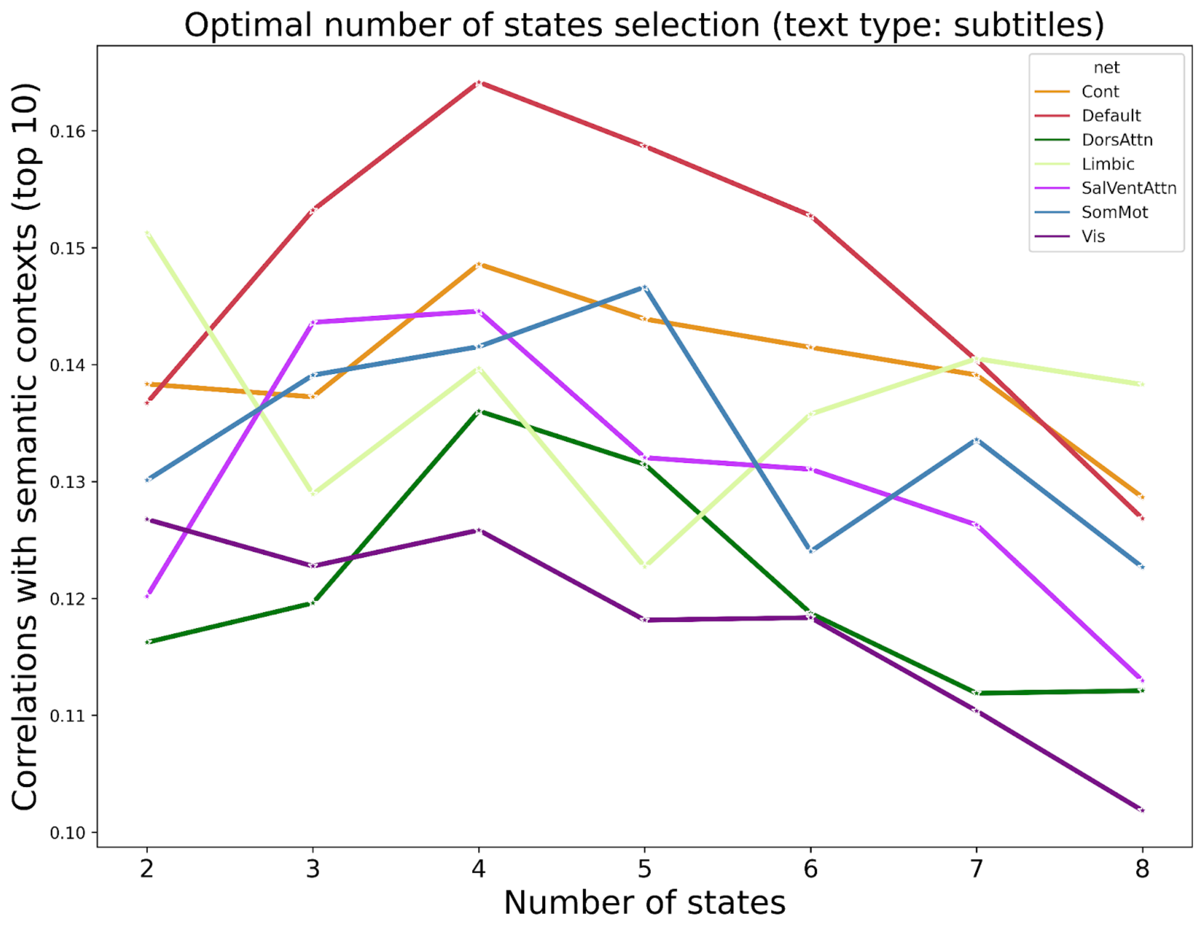

Supplementary Figure 12: Optimal number of states selected for seven different canonical networks using the same selection procedure as Supplemental Figure 1 (A). To determine the optimal number of states for the seven different canonical networks analyzed in Figure X, we used the same selection procedure as described in Supplementary Figure 1 (A). This involved identifying the number of states that produced the highest average value of the top 10 Pearson correlation links between the Hidden Markov Model (HMM) models' state presence and extracted semantic contexts, which indicated the closest alignment of the brain activity model with movie narrative features. Source data are provided as a Source Data file.

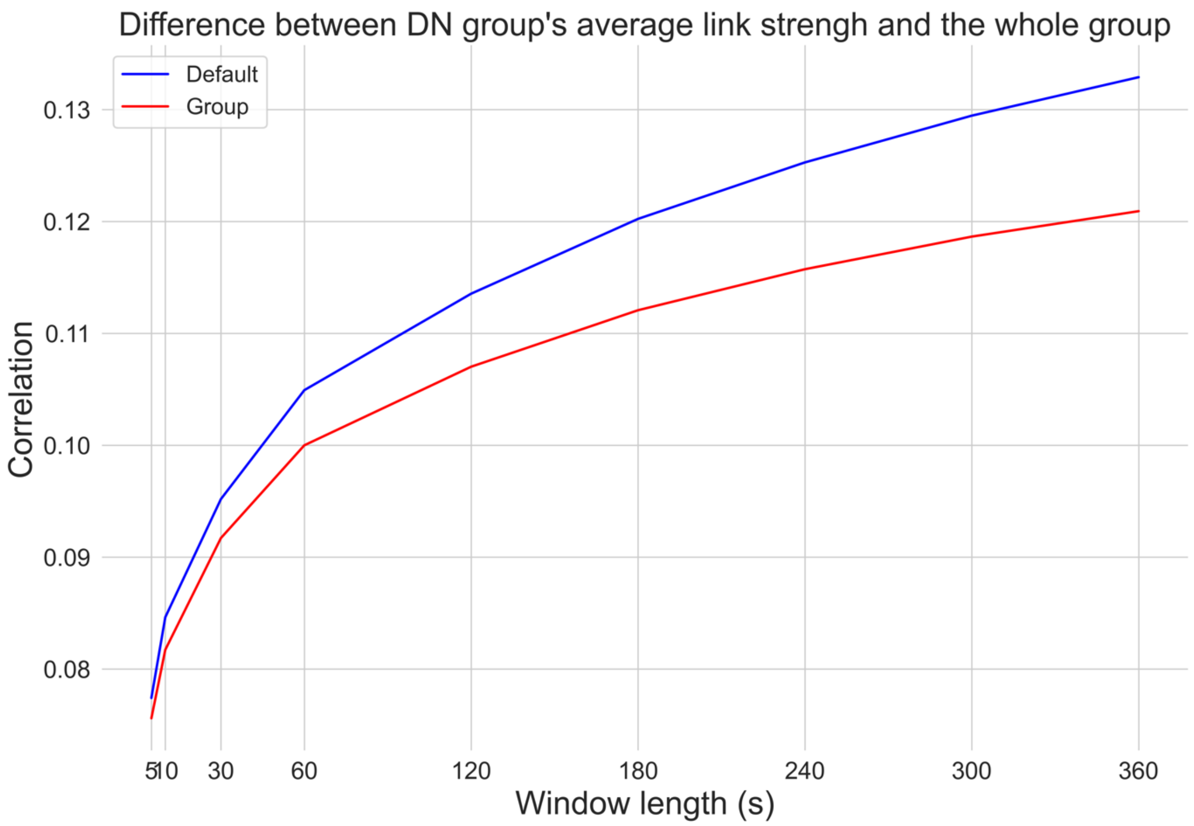

Supplementary Figure 13: Comparison of average link strength between DN group and the whole group. The figure shows a comparison between the average link strength of the DN group and the entire group. The x-axis represents the window length in seconds and the y-axis represents the correlation. We obtained new sets of semantic labels for different window lengths, and calculated the semantic brain link strength for all of our brain states using these new variables. We then calculated the average link strength for the Default Network (blue line) and for all 7 networks (orange line). The plot provides a visual representation of the difference in average link strength between the DN group and the entire group. Source data are provided as a Source Data file.

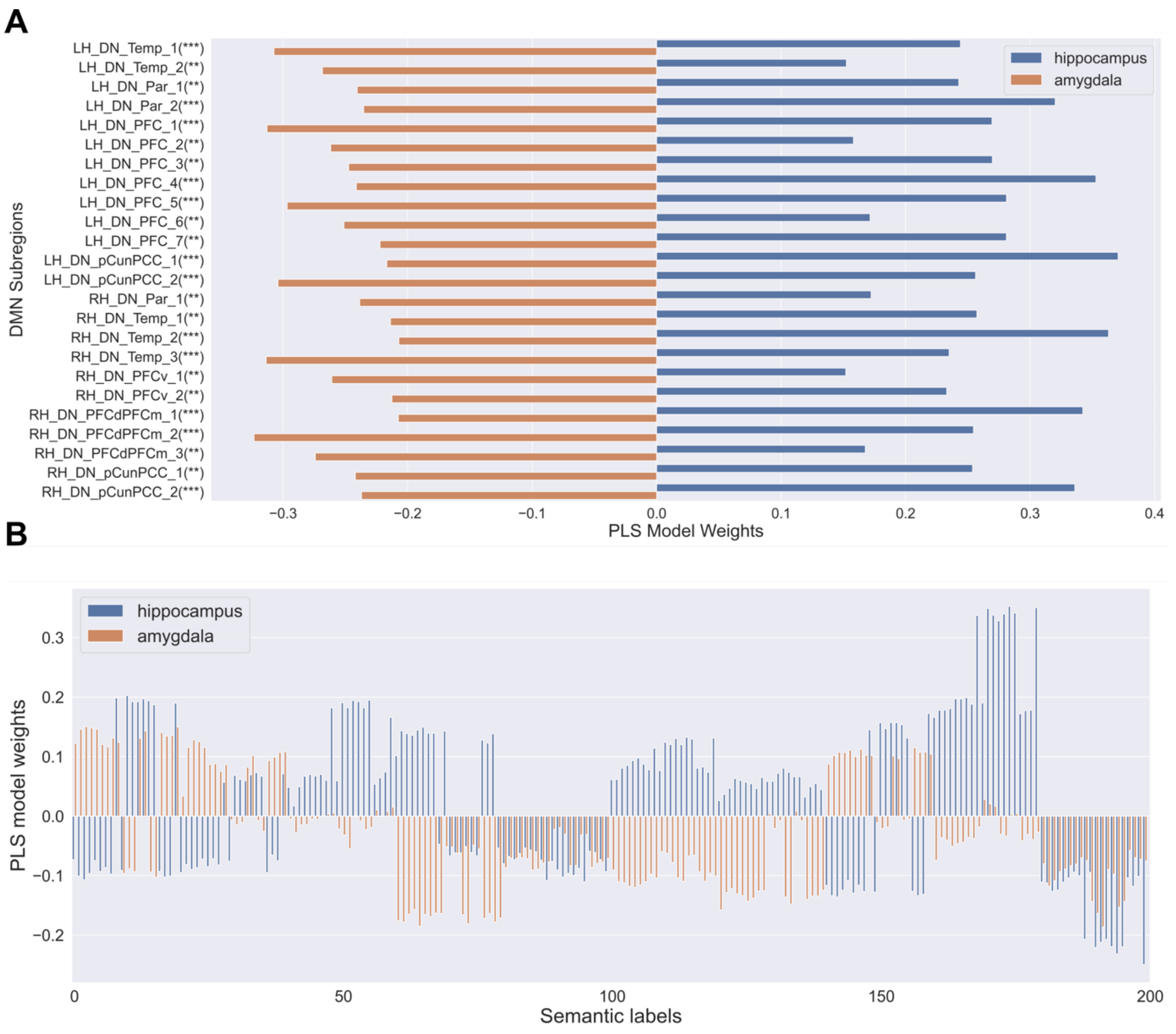

Supplementary Figure 14: Comparison of PLS model contributions between DN&AM and DN&HC models. (A) We present the comparison of loading parameters for the DN subregions between HC and AM groups using 20-fold cross-validation tests. Z-scored loading values from 20 partial datasets were compared for each subregion using a two-sample two-sided t-test. Significance levels are shown on the y-tick marks, with asterisks indicating p-values less than 0.05, 0.01, and 0.001. (B) The same procedure was applied to 200 semantic labels generated by NLP and statistically significant differences were found between the DN&HC and DN&AM groups, as indicated by p-values less than 0.001 for each pair of semantic labels using a two-sample two-sided t-test. The DN's subregion names are from Schaefer Yeo 100 atlas (Temp: temporal; Par: parietal; PFC: prefrontal cortex; pCunPCC: precuneus posterior cingulate cortex; PFCv/d/m: ventral/dorsal/medial prefrontal cortex;). The left and right hemispheres are denoted as LH and RH. Source data are provided as a Source Data file.

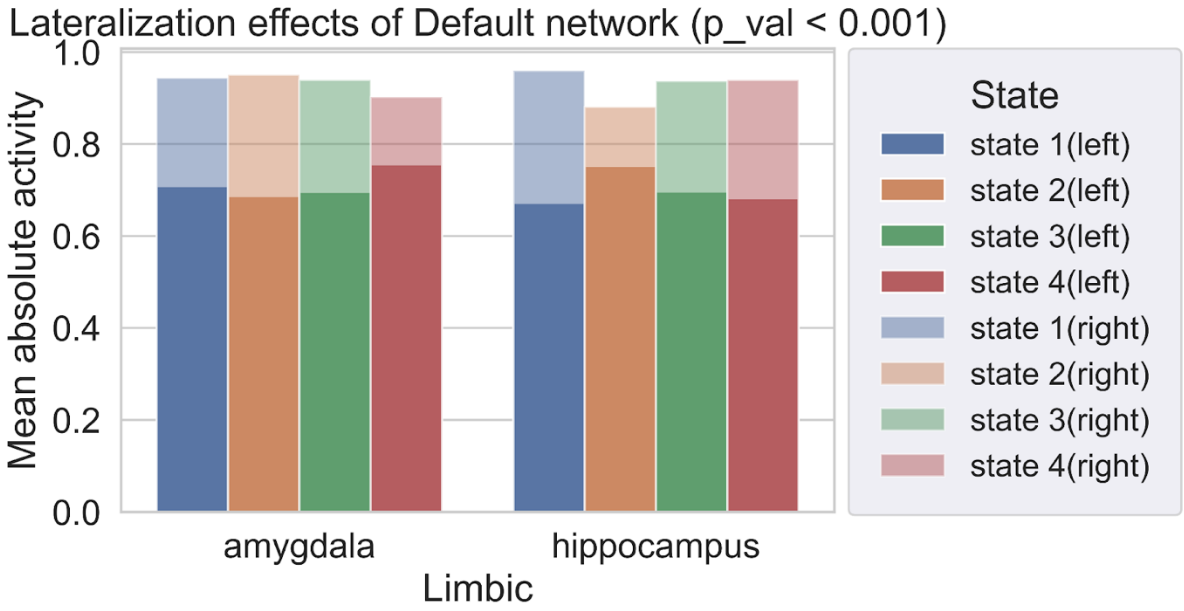

Supplementary Figure 15. Lateralization effects of the Default network. In the bar plot, we show the mean absolute activity across left and right hemisphere Default network (DN) subregions. The solid bars represent the left hemisphere, while the transparent ones represent the right hemisphere. Across four brain states and different subcortical partners, we consistently observe smaller activity in the left hemisphere compared to the right hemisphere. We performed a two-sample two sided t-test to test for group differences, and the results were significant (p-value < 0.001). Source data are provided as a Source Data file.

A

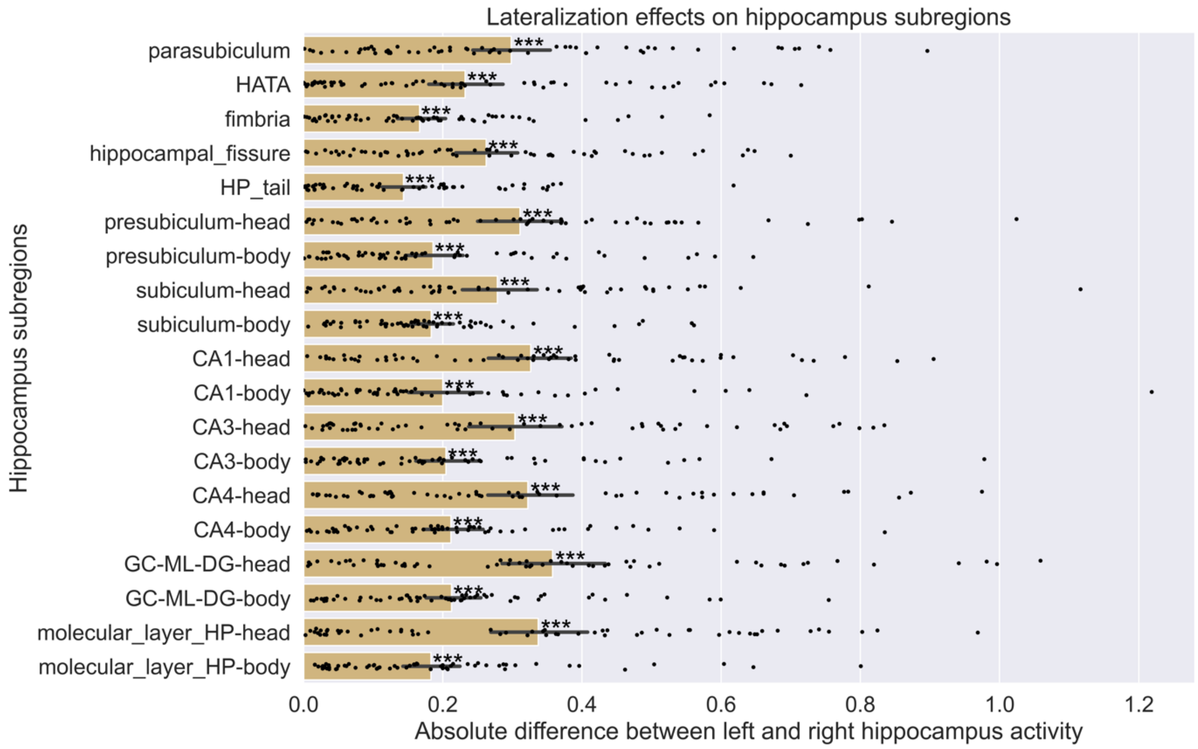

B

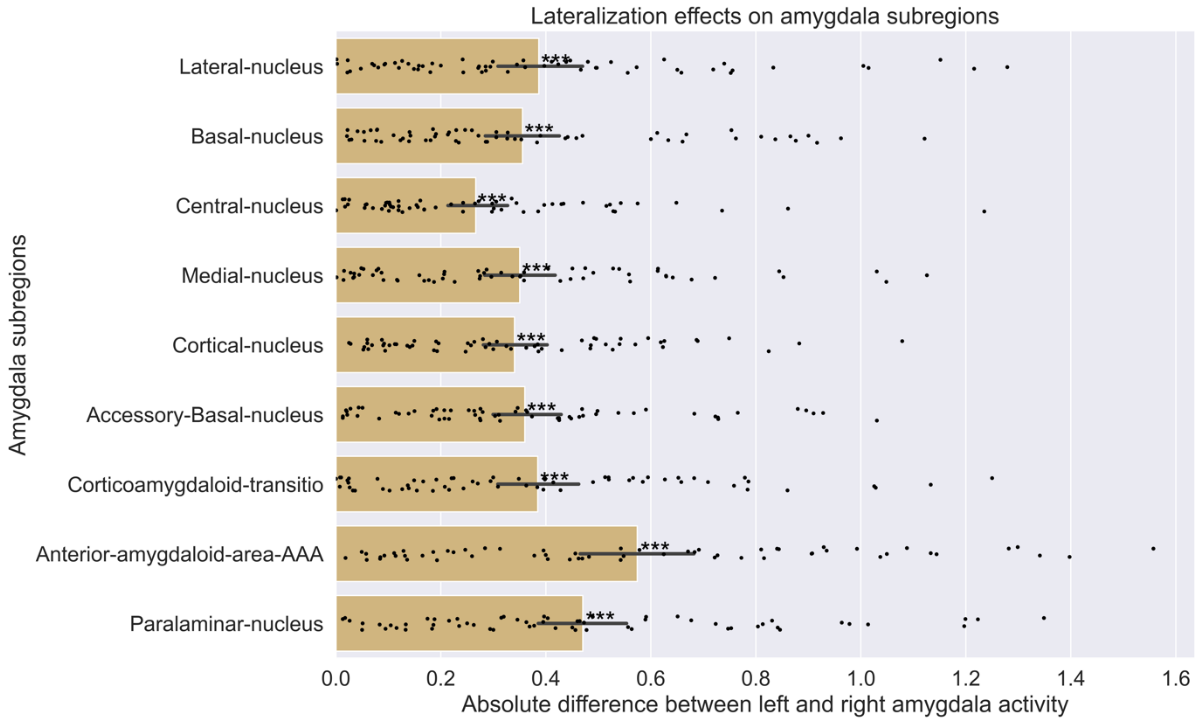

Supplementary Figure 16: Lateralization effects of hippocampal and amygdala subregions. Panel A) displays the name of hippocampal subregions on the y-axis, and panel B) displays the name of amygdalar subregions. Each bar represents the mean of the absolute difference in activity across the four brain states, calculated for 15 subjects (4 states for each subject, 60 in total). The error bars indicate the 95% confidence interval, and significance generated by one sample t test (if absolute difference is greater than zero) is denoted by *** (p-value < 0.001) for both subregions. CA: dentate gyrus and Cornu Ammonis, HATA:

hippocampal amygdala transition area; HP: hippocampus; ML: molecular layer; DG: granule cell layer of the dentate gyrus (GC-DG-ML). Source data are provided as a Source Data file. Source data are provided as a Source Data file.

Region-network combination	median	std
**FPC&AM**	14.95	7.26
**FPC&HC**	11.85	9.50
**DN&AM**	16.62	9.25
**DN&HC**	16.87	8.30
**DAN&AM**	11.58	6.40
**DAN&HC**	10.27	5.14
**LIM&AM**	9.29	11.96
**LIM&HC**	5.11	13.28
**VAN&AM**	15.11	6.40
**VAN&HC**	10.99	7.29
**SMN&AM**	11.18	5.56
**SMN&HC**	11.41	5.74
**VIS&AM**	10.12	3.54
**VIS&HC**	10.29	3.59

Supplementary Table 1: The table of model specific average dwell times across 14 region-network combinations. Short names for Schaefer-yeo networks: VIS: Visual network; SMN: somatomotor network; DAN: dorsal attention network; DN: default network; LIM: limbic network; VAN: salience and ventral attention network; FPC: Frontoparietal network. Source data are provided as a Source Data file.
