# Supplementary material for: The default network dominates neural responses to evolving movie stories
Source: Nat Commun. 2023 Jul 14;14:4197. doi: 10.1038/s41467-023-39862-y (PMC10349102; doi:10.1038/s41467-023-39862-y)
Supplement: Supplementary file 3 — Reporting Summary [file 41467_2023_39862_MOESM3_ESM.pdf]

nature portfolio

Corresponding author(s): Enning Yang

Last updated by author(s): May 16, 2023

Reporting Summary

Nature Portfolio wishes to improve the reproducibility of the work that we publish. This form provides structure for consistency and transparency in reporting. For further information on Nature Portfolio policies, see our Editorial Policies and the Editorial Policy Checklist.

Statistics

For all statistical analyses, confirm that the following items are present in the figure legend, table legend, main text, or Methods section.

n/a	Confirmed	
☐	☒	The exact sample size (*n*) for each experimental group/condition, given as a discrete number and unit of measurement
☐	☒	A statement on whether measurements were taken from distinct samples or whether the same sample was measured repeatedly
☐	☒	The statistical test(s) used AND whether they are one- or two-sided<br>*Only common tests should be described solely by name; describe more complex techniques in the Methods section.*
☐	☒	A description of all covariates tested
☒	☐	A description of any assumptions or corrections, such as tests of normality and adjustment for multiple comparisons
☐	☒	A full description of the statistical parameters including central tendency (e.g. means) or other basic estimates (e.g. regression coefficient) AND variation (e.g. standard deviation) or associated estimates of uncertainty (e.g. confidence intervals)
☐	☒	For null hypothesis testing, the test statistic (e.g. *F*, *t*, *r*) with confidence intervals, effect sizes, degrees of freedom and *P* value noted<br>*Give P values as exact values whenever suitable.*
☒	☐	For Bayesian analysis, information on the choice of priors and Markov chain Monte Carlo settings
☐	☒	For hierarchical and complex designs, identification of the appropriate level for tests and full reporting of outcomes
☐	☒	Estimates of effect sizes (e.g. Cohen's *d*, Pearson's *r*), indicating how they were calculated

Our web collection on statistics for biologists contains articles on many of the points above.

Software and code

Policy information about availability of computer code

Data collection	No software was used.

Data analysis	freesurfer == 7.1.1<br>python == 3.8.8<br>hmmlearn == 0.2.6<br>matplotlib == 3.3.4<br>nibabel == 3.2.1<br>nilearn == 0.9.0<br>nltk == 3.6.1<br>numpy == 1.20.1<br>pandas == 1.2.4<br>plotly == 5.1.0<br>scikit_learn == 1.0.2<br>scipy == 1.6.2<br>seaborn == 0.11.1<br>statannot == 0.2.3<br>utils == 1.0.1<br>wordcloud == 1.8.1

For manuscripts utilizing custom algorithms or software that are central to the research but not yet described in published literature, software must be made available to editors and reviewers. We strongly encourage code deposition in a community repository (e.g. GitHub). See the Nature Portfolio guidelines for submitting code & software for further information.

Data

Policy information about availability of data

All manuscripts must include a data availability statement. This statement should provide the following information, where applicable:

- Accession codes, unique identifiers, or web links for publicly available datasets
- A description of any restrictions on data availability
- For clinical datasets or third party data, please ensure that the statement adheres to our policy

Data availability:
The intermediate data generated in this study have been deposited in the Google Drive (https://drive.google.com/drive/folders/1Fq0XzNU0qN6bIFVhH3pBwXqPKOWznx6t?usp=sharing) database. The raw fMRI data, structural MRI, and annotations are available on Studyforrest's official website (https://www.studyforrest.org/).

Research involving human participants, their data, or biological material

Policy information about studies with human participants or human data. See also policy information about sex, gender (identity/presentation), and sexual orientation and race, ethnicity and racism.

Reporting on sex and gender	Our study includes participants of both sexes in the dataset; however, we did not conduct a sex-based analysis.
Reporting on race, ethnicity, or other socially relevant groupings	na
Population characteristics	15 subjects were recruited, with a mean age of 29.4 years (ranging from 21 to 39). Out of the recruited subjects, 6 were females.
Recruitment	We didn't have any new recruitment in this study.
Ethics oversight	We didn't have any new recruitment in this study.

Note that full information on the approval of the study protocol must also be provided in the manuscript.

Field-specific reporting

Please select the one below that is the best fit for your research. If you are not sure, read the appropriate sections before making your selection.

☒ Life sciences ☐ Behavioural & social sciences ☐ Ecological, evolutionary & environmental sciences

For a reference copy of the document with all sections, see nature.com/documents/nr-reporting-summary-flat.pdf

Life sciences study design

All studies must disclose on these points even when the disclosure is negative.

Sample size	We utilized the 15 subjects provided by the Studyforrest dataset, which consisted of over 50,000 individual brain scans.
Data exclusions	no data was excluded

Replication	We did not collect the data ourselves, so no replication experiment at the life science level was performed in this study. However, we did conduct a replication experiment for data analysis.
Randomization	We utilized permutation tests and cross-validation in our analysis.
Blinding	no

Reporting for specific materials, systems and methods

We require information from authors about some types of materials, experimental systems and methods used in many studies. Here, indicate whether each material, system or method listed is relevant to your study. If you are not sure if a list item applies to your research, read the appropriate section before selecting a response.

Materials & experimental systems

n/a	Involved in the study
☒	☐ Antibodies
☒	☐ Eukaryotic cell lines
☒	☐ Palaeontology and archaeology
☒	☐ Animals and other organisms
☒	☐ Clinical data
☒	☐ Dual use research of concern
☒	☐ Plants

Methods

n/a	Involved in the study
☒	☐ ChIP-seq
☒	☐ Flow cytometry
☐	☒ MRI-based neuroimaging

Magnetic resonance imaging

Experimental design

Design type	task
Design specifications	Each subject had a total of 3541 time points, and there were 8 trials per person.
Behavioral performance measures	no measure was used

Acquisition

Imaging type(s)	functional and structural
Field strength	3 T
Sequence & imaging parameters	The data utilized in this study was collected by the Studyforrest dataset.
Area of acquisition	The data utilized in this study was collected by the Studyforrest dataset.

Diffusion MRI ☐ Used ☒ Not used

Preprocessing

Preprocessing software	The data utilized in this study was collected by the Studyforrest dataset.
Normalization	The data utilized in this study was collected by the Studyforrest dataset.
Normalization template	The data utilized in this study was collected by the Studyforrest dataset.
Noise and artifact removal	The data utilized in this study was collected by the Studyforrest dataset.
Volume censoring	The data utilized in this study was collected by the Studyforrest dataset.

Statistical modeling & inference

Model type and settings	The data utilized in this study was collected by the Studyforrest dataset.
Effect(s) tested	The data utilized in this study was collected by the Studyforrest dataset.

Specify type of analysis: ☐ Whole brain ☒ ROI-based ☐ Both

Anatomical location(s): MNI 152

Statistic type for inference

(See Eklund et al. 2016)

The data utilized in this study was collected by the Studyforrest dataset.

Correction

The data utilized in this study was collected by the Studyforrest dataset.

Models & analysis

n/a	Involved in the study
☒	☐ Functional and/or effective connectivity
☒	☐ Graph analysis
☐	☒ Multivariate modeling or predictive analysis

Multivariate modeling and predictive analysis

We employed Principal Component Analysis (PCA), Hidden Markov Model (HMM), and Partial Least Squares (PLS) in our analysis.
